# Postoperative intralesional 5-fluorouracil therapy for early recurrence management of pterygium

**DOI:** 10.5935/0004-2749.20210058

**Published:** 2025-02-02

**Authors:** Magda Massae Hata Viveiros, Rodolfo de Lima Fachinelli, Larissa Lucas, Silvana Artioli Schellini

**Affiliations:** 1 Department of Ophthalmology, Faculdade de Medicina, Universidade Estadual Paulista “Júlio de Mesquita Filho”, Botucatu, SP, Brazil

Dear Editor,

The major concern with pterygium treatment is the high rate of recurrence (10%-80%), depending on the excision technique employed)^(1,2)^. Owing to the risk of recurrence, adjuvant therapies such as with mitomycin C (MMC) and 5-fluorouracil (5FU) have been recommended to reduce the proliferation of fibroblasts. Therapy with 5FU inhibits the synthesis of fibroblast DNA and RNA through the inhibition of the enzyme thymidylate synthetase; this treatment is more effective when performed during the synthesis phase of the cell cycle as it reduces the levels of cytokines and growth factors released after surgical trauma, which otherwise can lead to the recurrence of pterygium^(3)^.

Five-FU is a safe and effective agent useful in reducing the recurrence rates of primary and recurrent lesions. The treatment approach can be intralesional at 30 and 10 days before the surgery, topical or intralesional immediately after the removal of the lesion, or drops in the postoperative period^(4,5)^.

Recently, intralesional 5FU therapy has been shown to produce good outcomes when applied for early recurrence immediately when the lesion starts regrowing^(6-8)^. We applied this approach in 4 recurrent pterygiums that were operated with a sliding conjunctival flap and sutured with 7-0 absorbable synthetic braided suture (Vycril - Ethicon, São José dos Campos, SP, Brazil). At the end of the surgery, a subconjunctival injection of 0.2 mL of 25 mg/mL of 5FU (Roche, São Paulo, Brazil) was performed in the remaining body of the pterygium. After 30 days of the surgery, 3 eyes with recurrent grade-II^^9^^ pterygium received 0.2 mL of 25 mg/mL of 5FU (Roche, São Paulo, Brazil) intralesional subconjunctival injection and 1 eye with grade IV^(9)^ recurrence received 4 weekly injections using the same dose and concentration of 5-FU. Prophylactic antibiotics and steroid eyedrops 4 times a day were prescribed after the injection. There were no complications during the procedure, but patients complained of pain and discomfort during the injection. The same previous clinical appearance, with increased vascularity and thick conjunctiva was observed in all of our treated patients. Although the pterygium lesion has not diminished, no progress has been observed. After 180 days, all 4 treated eyes maintained the same clinical appearance and degree of recurrence without reducing the recurrent pterygium.

In conclusion, the intralesional postoperative use of 5FU in early recidivate pterygium is not effective in reducing the chances of recurrences.
